# Associations between quantitative measures of TDLU involution and breast tumor molecular subtypes among breast cancer cases in the Black Women’s Health Study: a case–case analysis

**DOI:** 10.1186/s13058-022-01577-1

**Published:** 2022-12-05

**Authors:** Brittny C. Davis Lynn, Brittany D. Lord, Renata Cora, Ruth M. Pfeiffer, Scott Lawrence, Gary Zirpoli, Traci N. Bethea, Julie R. Palmer, Gretchen L. Gierach

**Affiliations:** 1 Division of Cancer Epidemiology and Genetics, National Cancer Institute, 9609 Medical Center Drive, Rockville, MD USA; 2 Stamford, CT USA; 3 Molecular and Digital Pathology Laboratory, Leidos Biomedical Research, Inc., 9615 Medical Center Drive, Rockville, MD USA; 4 Slone Epidemiology Center, Boston University, 72 East Concord Street L-7, Boston, MA USA; 5 Office of Minority Health and Health Disparities Research, Georgetown Lombardi Comprehensive Cancer Center, 1000 New Jersey Ave SE, Washington, DC USA

**Keywords:** Triple-negative, Terminal duct lobular unit, Luminal A, Breast cancer risk factors, TDLU involution

## Abstract

**Background:**

Terminal duct lobular units (TDLUs) are the structures in the breast that give rise to most breast cancers. Previous work has shown that TDLU involution is inversely associated with TDLU metrics, such as TDLU count/100mm^2^, TDLU span (µm), and number of acini/TDLU, and that these metrics may be elevated in the normal breast tissue of women diagnosed with triple-negative (TN) compared with luminal A breast tumors. It is unknown whether this relationship exists in Black women, who have the highest incidence of TN breast cancer and the highest overall breast cancer mortality rate. We examined relationships between TDLU metrics and breast cancer molecular subtype among breast cancer cases in the Black Women’s Health Study (BWHS).

**Methods:**

We assessed quantitative TDLU metrics (TDLU count/100mm^2^, TDLU span (µm), and number of acini/TDLU) in digitized 247 hematoxylin and eosin-stained adjacent normal tissue sections from 223 BWHS breast cancer cases, including 65 triple negative (TN) cancers (estrogen receptor (ER) negative, progesterone receptor (PR) negative, human epidermal growth factor-2 (HER2) negative) and 158 luminal A cancers (ER positive, HER2 negative). We evaluated associations of least square mean TDLU metrics adjusted for age and body mass index (BMI) with patient and clinical characteristics. In logistic regression models, we evaluated associations between TDLU metrics and breast cancer subtype, adjusting for age, BMI, and tumor size.

**Results:**

Older age and higher BMI were associated with lower TDLU metrics and larger tumor size and lymph node invasion with higher TDLU metrics. The odds of TN compared with luminal A breast cancer increased with increasing tertiles of TDLU metrics, with odds ratios (95% confidence intervals) for tertile 3 versus tertile 1 of 2.18 (0.99, 4.79), 2.77 (1.07, 7.16), and 1.77 (0.79, 3.98) for TDLU count, TDLU span, and acini count/TDLU, respectively.

**Conclusion:**

Associations of TDLU metrics with breast cancer subtypes in the BWHS are consistent with previous studies of White and Asian women, demonstrating reduced TDLU involution in TN compared with luminal A breast cancers. Further investigation is needed to understand the factors that influence TDLU involution and the mechanisms that mediate TDLU involution and breast cancer subtype.

**Supplementary Information:**

The online version contains supplementary material available at 10.1186/s13058-022-01577-1.

## Background

Triple negative (TN) breast cancer, an early-age onset and aggressive form, has the highest incidence in Black women among US women [1]. Studies have suggested that environmental and social factors may contribute to the higher risk of TN breast cancer among Black women compared with other racial and ethnic groups [2–5]. Additionally, certain reproductive factors, such as high parity with no breastfeeding, are more common in Black women and have been shown to increase risk for TN breast cancer [6, 7]. However, it is unknown what molecular mechanisms might underlie these relationships.

Recent work has shown that histological measures of terminal duct lobular unit (TDLU) involution in adjacent normal breast tissues are associated with TN breast cancer risk relative to luminal A breast cancer in Chinese and European women [8–10]. Characterization of TDLU involution in Black women with breast cancer has been largely unexplored. TDLUs are the anatomical structures in the breast responsible for producing milk during pregnancy and postpartum, as well as the structures from which most breast cancers arise [11]. TDLU involution, the process by which these structures ablate/disappear, occurs during the postpartum period and with physiological aging [12].

Higher levels of quantitative TDLU metrics (number of TDLUs per 100mm^2^, TDLU span/diameter (microns), and number of acini (substructures) per TDLU) have been shown to be inversely associated with TDLU involution and positively associated with breast cancer risk among women diagnosed with benign breast disease (BBD) [12–15]. Previous studies have also shown that TDLU metrics are associated with certain breast cancer risk factors, for example with a lower number of TDLUs observed in nulliparous women as compared with parous women [9, 14].

The aim of this study was to evaluate associations of quantitative measures of TDLU involution with breast cancer molecular subtype among women diagnosed with breast cancer in the Black Women’s Health Study (BWHS), and to examine associations of TDLU measures with reproductive risk factors. Understanding these relationships in Black women, who are at increased risk for TN breast cancer, may be important for better understanding the etiologic and pathologic heterogeneity of breast cancer.

## Methods

### Study population and breast tissue collection

The Black Women’s Health Study (BWHS) is a prospective cohort that enrolled 59,000 self-identified Black women, ages 21–69 in 1995 [16] and has followed them by biennial questionnaire since that time. Study participants who had been diagnosed with breast cancer from 1999 to 2018 were asked to provide consent for study investigators to obtain their archived breast tumor tissue for use in breast cancer research; 54% of women with breast cancer returned a signed consent and breast tumor samples were successfully retrieved for 75% of those women, from multiple hospitals across the USA. In total, breast tumor tissue was obtained for 849 women with luminal A (ER positive and HER2 negative) and 324 women with TN breast cancer (ER negative, PR negative, and HER2 negative). Although normal adjacent breast tissue was requested at the same time, for many cases, there was insufficient material for examination. Among those, 1,173 cases with breast tumor tissue, normal adjacent tissue samples were obtained for 158 women with luminal A breast cancer and 65 women with TN breast cancer.

### Evaluation of TDLU involution

Hematoxylin and eosin (H&E)-stained slides of adjacent normal tissues were shipped to the Molecular and Digital Pathology Laboratory at the National Cancer Institute, digitized at 40X magnification (Leica AT2) and prepared for viewing and annotation with HALO Link (Indica Labs, Albuquerque, New Mexico) [17].

Digitized H&E-stained whole slide images were reviewed by a single expert reviewer (RC) masked to other participant data to estimate percentage of fat and the presence of normal TDLUs. Tissue sections containing cancer were not evaluated. TDLUs were not evaluated if at least half the acini were dilated 2–3 times the normal diameter or if metaplastic changes involved at least half the acini. Sections with the presence of normal TDLUs were then analyzed for standardized measures of TDLU involution, including number of TDLUs per 100mm^2^. For TDLU span (microns) and number of acini per TDLU, up to 10 normal TDLUs were reviewed sequentially as follows: 1) TDLU span was measured with an electronic ruler (microns) and 2) acini counts/TDLU were assessed visually in categories (1 = 2–10, 2 = 11–20, 3 = 21–30, 4 = 31–50, 5 = 50–100, 6 = > 100), to provide stable representative measures of TDLU involution. For acini counts/TDLU and TDLU span measures, we used the median of the values obtained across the multiple TDLUs measured for each woman. These quantitative TDLU metrics have been found to be reliable and correlated with qualitative assessments of TDLU involution as previously described [14].

The analysis was restricted to women diagnosed with luminal A (n = 158) and TN (n = 65) breast cancers to remain consistent with previous analyses of TDLU involution measures and breast cancer subtypes [10] in other populations. For the 223 women included in the current study, there were 253 total slides available for analysis, as some women had more than one slide available (2 slides, n = 9; 3 slides, n = 5; 4 slides, n = 2; > 5 slides, n = 2). Six slides (4 from luminal A cases and 2 from TN cases) were excluded because they included cancer, leaving 247 slides for the analysis.

### Statistical analysis

Patient characteristics were compared using Chi-squared tests for categorical variables or student’s t-test for continuous variables. Adjusted least square means of each TDLU involution measure by participant characteristics were estimated using linear regression, adjusting for categorical age (≤ 55 or > 55 years) and body mass index (BMI, kg/m^2^) (< 25, 25–29, ≥ 30), and stratifying by breast cancer molecular subtype. Logistic regression was used to estimate associations (odds ratios [ORs] and 95% confidence intervals [CIs]) between TDLU involution measures and breast cancer subtypes (TN vs. luminal A). Tertile levels of standardized TDLU involution measures were created based on the distribution in the luminal A cases. Potential confounding factors were included as adjustment variables if the *p* value/trend was < 0.05 based on Chi-square tests for their associations with at least one TDLU involution measure and breast cancer subtype. To conserve degrees of freedom, we adjusted for age using a dichotomous variable and BMI and tumor size using an ordinal trend. Sensitivity analysis with additional adjustment for percent fat on the slide was also performed.

The R package “geepack” [18] was used to obtain *p* values and variance estimates that account for the multiple observations (whole slide images) for some individuals. All statistical tests were two-sided, and a *p* value < 0.05 was considered statistically significant. R version 3.6.1 was used for all calculations.

## Results

### Population characteristics

Table 1 details patient characteristics by breast tumor molecular subtype. We observed significant differences by age at diagnosis (*p* = 0.04), BMI (*p* = 0.05), tumor size (*p* = 0.001), and tumor grade (*p* < 0.001). Compared with luminal A cases, the TN cases were more likely to be younger (≤ 55 years of age) at diagnosis and to have poorly differentiated and larger (> 2 cm) tumors (Table 1). Participant characteristics were similar between all luminal A and TN cases in the BWHS and the subset included in this study.

**Table 1:** Characteristics of BWHS participants with adjacent normal breast tissue, according to molecular subtype

Variable	Luminal A (n = 158)		Triple-negative (n = 65)		*p* value^^^
	n	%	n	%	
*Age at diagnosis*					
mean (range)	53.9	29–82	52.7	35–76	0.34^#^
29–55	86	54.4	46	70.8	**0.04**
56–82	72	45.6	19	29.2	
*Smoking*					
Never	90	57.3	39	60.9	0.77
Former	50	31.8	20	31.3	
Current	17	10.8	5	7.8	
Missing*	1		1		
*BMI (kg/m*^*2*^*)*					
18.7–< 25	34	21.7	15	23.1	**0.05**
25–29	42	26.8	27	41.5	
≥ 30–57.1	81	51.6	23	35.4	
Missing	1		1		
*Age at menarche*					
< 12	51	32.3	25	38.5	0.57
12–13	90	57	32	49.2	
≥ 14	17	10.8	8	12.3	
*Parity*					
Nulliparous	30	19	9	13.8	0.47
Parous	128	81	56	86.2	
*Live births*					
Nulliparous	30	19	9	13.8	0.17
1	43	27.2	15	23.1	
2	46	29.1	29	44.6	
3+	39	24.7	12	18.5	
*Age at first birth (among parous)*					
< 20 years	38	30.2	14	25	0.65
20–24 years	35	27.8	19	33.9	
≥ 25 years	53	42.1	23	41.1	
Missing	2		0		
*Years since last birth (among parous)*					
< 5 years	3	2.4	0	0	0.27**
5–9 years	10	8	3	5.4	
10–14 years	9	7.2	8	14.3	
15–19 years	17	13.6	4	7.1	
20+ years	86	68.8	41	73.2	
Missing	3		0		
*Breastfeeding (among parous)*					
Never	64	50.8	24	43.6	0.3
Ever	62	49.2	31	56.4	
Missing	2		1		
*Menopausal status*					
Pre-menopausal	60	38	26	40	1
Post-menopausal	81	51.3	27	41.5	
Unknown	17	10.8	12	18.5	
*Age at menopause (among post-menopausal)*					
< 50	47	48	14	35.9	0.83
≥ 50	33	33.7	12	30.8	
Unknown	18	18.4	13	33.3	
*History of benign breast disease*					
No	101	63.9	39	60	0.69
Yes	57	36.1	26	40	
*First degree family history of breast cancer*					
No	134	84.8	57	87.7	0.73
Yes	24	15.2	8	12.3	
*Tumor size*					
≤ 2 cm	119	77.3	34	54	**0.001**
> 2 cm	35	22.7	29	46	
Missing	4		2		
*Tumor grade*					
Well/moderately differentiated	122	79.7	9	14.5	< 0.001**
Poorly differentiated	31	20.3	53	85.5	
Missing	5		3		
*Lymph node invasion*					
Negative	98	65.3	37	63.8	0.96
Positive	52	34.7	21	36.2	
missing	8		7		

^^^*p* value obtained from Chi-squared test

^#^*p* value obtained from Student's t-test

*Missing excluded from percentages and testing

**Fisher's exact test performed for variables with low cell counts

*P*-values < 0.05 are noted in bold

### Associations between TDLU metrics and breast cancer subtype

Table 2 shows the univariate and multivariable associations between TDLU involution measures and breast cancer subtype. TDLUs were observed in the adjacent normal tissue blocks in 93% of luminal A cases and 99% of TN cases. Compared with luminal A cases, TN cases were more likely to have higher TDLU count, median TDLU span, and acini count/TDLU (i.e., less TDLU involution). Odds of TN breast cancer were 2.44 (95% CI 1.14, 5.22), 2.98 (95% CI 1.21, 7.36), and 1.93 (95% CI 0.88, 4.22) for tertile 3 compared to tertile 1 for TDLU count, TDLU span, and acini count/TDLU, respectively, after adjustment for age and BMI. After further adjustment for tumor size, observed patterns of association were similar but only the relationship between median TDLU span and breast cancer subtype remained statistically significant with ORs of 3.17 (95% CI 1.32, 7.61) and 2.77 (95% CI 1.07, 7.16) for tertiles 2 and 3, respectively, compared with tertile 1. Associations remained unchanged in a sensitivity analysis that additionally adjusted for percent fat observed on the H&E (Additional file 1: Table 1).

**Table 2:** Association between TDLU measures and breast cancer subtype among 247 adjacent normal breast tissue slides

TDLU measure			Unadjusted	Adjusted*	Adjusted**
	Luminal A (n = 174)	TNBC (n = 73)	TNBC versus Lum. A	TNBC versus Lum. A	TNBC versus Lum. A
	n (%)	n (%)	OR (95% CI)	OR (95% CI)	OR (95% CI)
*TDLUs present*					
Yes	161 (93%)	72 (99%)	Ref	Ref	Ref
No	13 (7%)	1 (1%)	0.17 (0.02, 1.34)	0.20 (0.03, 1.54)	0.16 (0.02, 1.20)
*TDLU count/100 mm*^*2*^					
Tertile 1	58 (33%)	13 (18%)	Ref	Ref	Ref
Tertile 2	57 (33%)	23 (32%)	1.80 (0.84, 3.86)	1.81 (0.85, 3.85)	1.60 (0.73, 3.51)
Tertile 3	59 (34%)	37 (51%)	**2.80 (1.32, 5.92)**	2.44 (1.14, 5.22)	2.18 (0.99, 4.79)
			P-trend **0.01**	P-trend **0.02**	P-trend 0.06
Median TDLU span (µm)***					
Tertile 1	53 (33%)	9 (13%)	Ref	Ref	Ref
Tertile 2	54 (34%)	30 (42%)	3.27 (1.41, 7.57)	3.11 (1.34, 7.19)	3.17 (1.32, 7.61)
Tertile 3	54 (34%)	33 (46%)	**3.60 (1.51, 8.60)**	**2.98 (1.21, 7.36)**	**2.77 (1.07, 7.16)**
			P-trend **0.01**	P-trend **0.02**	P-trend 0.05
Median acini count/TDLU***					
Tertile1	64 (40%)	18 (25%)	Ref	Ref	Ref
Tertile2	49 (30%)	24 (33%)	1.74 (0.83, 3.66)	1.61 (0.77, 3.36)	1.46 (0.70, 3.07)
Tertile3	48 (30%)	30 (42%)	2.22 (1.06, 4.68)	1.93 (0.88, 4.22)	1.77 (0.79, 3.98)
			P-trend **0.03**	P-trend 0.10	P-trend 0.17

Cut points based on distribution in Luminal A cases. TDLU count tertile cutpoints: T1 ≤ 4.81, T2 > 4.81 and  ≤ 15.84, T3 > 15.84; Median TDLU span: T1 ≤ 394.7, T2 > 394.7 and ≤ 600.0, T3 > 600.0; Median acini count/TDLU: T1 ≤ 6, T2 > 6 and ≤ 15.5, T3 > 15.5

*Logistic regression models adjusted for categorical age and BMI fitted with a trend coding

**Adjusted for categorical age, BMI, and tumor size fitted with a trend coding

***Among women with TDLUs

*P*-values < 0.05 are noted in bold

### Associations of standardized TDLU involution metrics with breast cancer risk factors and with breast cancer subtype

The TDLU involution measures were strongly and positively correlated with each other (Additional file 1: Table 2). Table 3 shows adjusted least square mean TDLU involution measures by participant characteristics within breast cancer subtype (adjusted for age and BMI). Many associations between TDLU metrics and breast cancer risk factors were statistically significant only among luminal A cases. Among luminal A cases: all TDLU metrics were inversely associated with age (*p* < 0.01); TDLU span and acini count/TDLU metrics were lower for former and current smokers compared with never smokers (*p* < 0.01 and *p* = 0.01, respectively); TDLU count was significantly increased among parous women compared with nulliparous women (*p* = 0.01) and among parous women whose last birth was within the last 20 years as compared with parous women whose last birth was 20 + years ago (*p* < 0.01). All TDLU measures were lower among post-menopausal women as compared with pre-menopausal women (*p* < 0.01 for all), a pattern that was observed but not statistically significant in TN cases. For both luminal A and TN cases, TDLU measures decreased with increasing BMI.

**Table 3:** Adjusted least square mean TDLU measurements in 247 adjacent normal tissues* by participant characteristics

Variable	Luminal A (n = 174)						Triple Negative (n = 73)					
	Measures of TDLU Involution						Measures of TDLU Involution					
	TDLU count/100mm^2^		Median TDLU span (microns)		Median number of acini/TDLU		TDLU count/100mm^2^		Median TDLU span (microns)		Median number of acini/TDLU	
	Mean (CI)	*p *value^	Mean (CI)	*p *value	Mean (CI)	*p *value	Mean (CI)	*p *value	Mean (CI)	*p *value	Mean (CI)	*p *value
*Age at diagnosis*												
≤ 55	23.7 (19.1, 28.3)		634.2 (585.6, 682.9)		24.6 (21.1, 28.2)		25.5 (19.3, 31.6)		612.6 (555.2, 670)		20.8 (16.5, 25.1)	
> 55	10.5 (5.2, 15.8)	** < 0.01**	447.6 (390.4, 504.7)	** < 0.01**	13.4 (9.2, 17.6)	** < 0.01**	22.2 (11.9, 32.4)	0.58	629.6 (535.2, 724)	0.75	23.2 (16, 30.3)	0.57
Smoking**												
Never	19.8 (15.3, 24.3)		568.6 (521.4, 615.8)		21.8 (18.4, 25.3)		24.1 (16.7, 31.5)		632.5 (562.5, 702.6)		23 (17.8, 28.3)	
Former	14 (7.6, 20.5)		538.6 (470.4, 606.8)		16.5 (11.4, 21.5)		20.2 (10.2, 30.2)		620.3 (526.6, 714.1)		20.6 (13.6, 27.7)	
Current	11.1 (0.8, 21.3)	0.12	415.5 (308.7, 522.2)	** < 0.01**	10.7 (2.8, 18.6)	**0.01**	41.4 (20.5, 62.2)	0.11	567.3 (371.3, 763.2)	0.52	23.9 (9.2, 38.5)	0.91
*BMI (kg/m*^*2*^)**												
< 25	18.1 (11.2, 24.9)		603.1 (531.2, 674.9)		24.2 (18.9, 29.5)		29.8 (18.4, 41.1)		700.6 (595.6, 805.5)		29.9 (22, 37.8)	
25–29	16.9 (10.2, 23.6)		510.2 (439.8, 580.6)		18.6 (13.4, 23.7)		26.5 (18.4, 34.6)		599 (524.2, 673.8)		19.9 (14.3, 25.5)	
≥ 30	16.4 (11.5, 21.2)	0.68	509.4 (456.7, 562.1)	**0.04**	14.2 (10.3, 18.1)	** < 0.01**	15.1 (5.6, 24.5)	**0.04**	563.7 (475.3, 652.2)	**0.04**	16.1 (9.5, 22.8)	** < 0.01**
Age at menarche**												
< 12	18.3 (11.7, 24.9)		511.2 (441.8, 580.5)		20.7 (15.6, 25.9)		31.4 (22.3, 40.5)		677.8 (591.6, 763.9)		26.5 (20.1, 33)	
12–13	17.4 (12.9, 21.8)		559.3 (511.7, 606.8)		18.3 (14.8, 21.8)		19.4 (10.9, 27.8)		609.3 (530.5, 688.1)		21.8 (15.9, 27.7)	
≥ 14	11.9 (1, 22.8)	0.31	509.4 (394, 624.7)	0.98	18.5 (10, 27.1)	0.66	19.9 (5.3, 34.4)	0.09	548.3 (411.8, 684.9)	0.13	14.5 (4.3, 24.7)	0.06
*Parity*												
Nulliparous	7.8 (-0.2, 15.8)		490.5 (398, 583)		18 (11.1, 24.8)		18.3 (3.6, 33.1)		557.8 (421.8, 693.8)		21.4 (11.1, 31.7)	
Parous	19.1 (15.2, 22.9)	**0.01**	550.1 (509.1, 591)	0.24	19.2 (16.2, 22.2)	0.74	25 (18.2, 31.7)	0.41	634.6 (572.5, 696.7)	0.31	22.1 (17.4, 26.8)	0.91
Live births**												
1	21.2 (14.3, 28.1)		563 (489.7, 636.2)		23 (17.6, 28.4)		33.8 (22.1, 45.6)		643.6 (530.6, 756.6)		24.3 (15.7, 32.9)	
2	16.2 (10, 22.4)		516.1 (450.7, 581.5)		17.7 (12.9, 22.5)		23.8 (15.4, 32.1)		652.1 (573.8, 730.3)		22.5 (16.6, 28.5)	
3+	20.4 (13.8, 26.9)	0.90	577.5 (507.2, 647.8)	0.72	17.5 (12.3, 22.6)	0.14	19 (6.1, 31.9)	0.08	578.7 (457.4, 700.1)	0.36	18.8 (9.5, 28)	0.64
Age at first birth (among parous)**												
< 20 years	16.5 (9.6, 23.4)		551.8 (486.2, 617.3)		16.2 (11.4, 21)		27.5 (15.3, 39.6)		708.5 (602.9, 814)		27 (19, 35)	
20–24 years	14.1 (6, 22.2)		539.4 (458.2, 620.6)		15.7 (9.8, 21.6)		16.7 (6.5, 26.8)		575.1 (486.1, 664.1)		16.9 (10.2, 23.7)	
≥ 25 years	23 (16.6, 29.5)	0.17	532.9 (471.9, 593.8)	0.68	22.3 (17.9, 26.8)	0.07	28.8 (19.1, 38.5)	0.85	644.9 (560.5, 729.4)	0.29	20.9 (14.5, 27.3)	0.18
*Years since last birth (among parous women)*												
< 20 years	31.0 (22.9, 39.2)		638.7 (560.6, 716.7)		24.2 (18.3, 30.0)		32.6 (20.2, 45.0)		657.0 (546.4, 767.6)		20.6 (12.2, 29.1)	
20+ years	11.8 (6.3, 17.3)	** < 0.01**	489.9 (437.6, 542.1)	**0.01**	15.7 (11.7, 19.6)	**0.03**	21.4 (13.9, 28.8)	0.1	625.2 (558.8, 691.5)	0.6	20.7 (15.7, 25.8)	0.98
*Breastfeeding (among parous)*												
Never	18.6 (12.5, 24.7)		570.6 (512, 629.1)		19.9 (15.5, 24.3)		20.2 (11, 29.3)		605.8 (523.9, 687.8)		20 (13.7, 26.3)	
Ever	19.3 (13.5, 25.1)	0.86	530.5 (474.9, 586)	0.32	18.7 (14.5, 22.8)	0.68	26.7 (17.6, 35.7)	0.28	660 (580.3, 739.7)	0.31	21.4 (15.3, 27.5)	0.74
*Menopausal status*												
Pre-meno	28.5 (21.1, 35.9)		674.6 (594.4, 754.8)		28.6 (22.7, 34.4)		30 (20, 40)		633.6 (538, 729.2)		25.4 (18.8, 32.1)	
Post-meno	9.3 (3.1, 15.4)	** < 0.01**	453.5 (386.2, 520.8)	** < 0.01**	12 (7.1, 17)	** < 0.01**	21.1 (12.4, 29.7)	0.19	609.3 (525.2, 693.4)	0.71	19.9 (14, 25.7)	0.22
*History of benign breast disease*												
No	15.7 (11.3, 20.1)		536 (488.9, 583.1)		18 (14.6, 21.5)		26.2 (18.8, 33.7)		626 (556.3, 695.7)		23.2 (18, 28.4)	
Yes	19.7 (13.7, 25.7)	0.28	549.9 (486.6, 613.1)	0.73	20.8 (16.1, 25.4)	0.35	20.3 (11.7, 29)	0.27	614.2 (533.5, 694.9)	0.81	20.3 (14.2, 26.3)	0.43
*Family history*												
No	17.3 (13.4, 21.2)		537.5 (495.7, 579.3)		19.1 (16.1, 22.2)		23 (16.6, 29.4)		620.3 (560.6, 679.9)		22 (17.5, 26.5)	
Yes	16.1 (7.2, 25.1)	0.82	557.4 (465.7, 649.2)	0.7	18.4 (11.6, 25.2)	0.84	28.8 (15.2, 42.5)	0.41	626.2 (499.6, 752.8)	0.93	22 (12.4, 31.5)	1
*Tumor size*												
≤ 2 cm	15.1 (11.4, 18.9)		530.4 (489, 571.7)		18.1 (15.1, 21.2)		25 (17.3, 32.7)		628.8 (557.1, 700.5)		22.5 (17.2, 27.9)	
> 2 cm	23.5 (16.2, 30.9)	**0.04**	559.5 (477.1, 642)	0.52	20.9 (14.8, 27)	0.41	23.2 (14.3, 32)	0.74	611.3 (528.2, 694.4)	0.73	21.7 (15.5, 27.9)	0.82
*Tumor grade*												
Well/moderately differentiated	17.8 (13.7, 22)		533.9 (490.1, 577.7)		20.4 (17.2, 23.6)		9.5 (-5.5, 24.5)		492 (354.1, 630)		13 (2.5, 23.5)	
Poorly differentiated	16.1 (7.5, 24.6)	0.71	539.3 (448.5, 630.1)	0.92	17.8 (11.1, 24.5)	0.48	25.7 (19.2, 32.2)	**0.05**	635.1 (574.8, 695.3)	0.06	23.4 (18.9, 28)	0.07
*Lymph node invasion*												
Negative	12.5 (8.5, 16.6)		505.3 (459.5, 551.1)		16 (12.8, 19.3)		21.6 (14.2, 28.9)		614.2 (544.5, 683.8)		19.9 (14.9, 24.9)	
Positive	23.6 (18, 29.2)	** < 0.01**	590 (527.1, 653)	**0.03**	23.1 (18.6, 27.5)	**0.01**	32.5 (22, 43.1)	0.07	659.1 (559.9, 758.2)	0.43	30.2 (23.1, 37.3)	**0.01**

All models adjusted for categorical age and BMI

^^^*p* values obtained from Student's T test

*Some women had more than one slide available for analysis (see “Methods”)

**Assessed using a p-trend test

*P*-values < 0.05 are noted in bold

Among luminal A cases, TDLU count was elevated for those with larger (> 2 cm: 23.5/100 mm^2^) versus smaller (≤ 2 cm: 15.1/100 mm^2^) tumors (*p* = 0.04). Among TN cases, all TDLU measures increased with increasing tumor grade, but the association was statistically significant only for TDLU count (*p* = 0.05). Among both case groups, all TDLU measures tended to be higher with positive lymph node invasion.

## Discussion

In this study of TDLU involution in adjacent normal tissues from Black women diagnosed with luminal A and TN breast cancers, we found that women diagnosed with TN breast cancer had reduced TDLU involution (i.e., higher TDLU count, larger TDLU span, and greater acini/TDLU) compared with women diagnosed with luminal A breast cancer. We also observed associations between TDLU involution measures and several participant and clinical characteristics. Prior work has demonstrated relationships between TDLU involution, breast cancer risk factors, and breast cancer development in European and Asian populations [9, 10]. Our findings in Black women suggest that TDLU involution may similarly play a substantial role in the etiologic heterogeneity of breast carcinogenesis and in the subsequent development of subtype-specific molecular features of breast tumors. These similarities in TDLU involution patterns across population groups are suggestive of common biological mechanisms that may underlie these relationships.

Our findings of decreased TDLU involution in TN breast cancer cases as compared with luminal A cases are consistent with previous studies examining TDLU involution measures in European and Chinese breast cancer patients. In particular, among European women diagnosed with breast cancer, the core basal phenotype (CBP; ER-negative, PR-negative, HER2-negative, cytokeratin 5-positive and/or epidermal growth factor receptor-positive) was associated with a greater average number of acini per TDLU and larger average TDLU diameter compared with luminal A cancers [10]. Likewise, among Chinese women diagnosed with breast cancer, increased acini counts were observed for those diagnosed with TN/CBP cancer compared with luminal A cancer [9]. A more recent study in healthy women investigating the relationship between genetic ancestry and TDLU involution has shown that women of African ancestry experience significantly decreased TDLU involution compared to women of European ancestry, potentially contributing to the etiologic heterogeneity of breast carcinogenesis experienced by these different racial groups [19]. Ours is the first study to compare TDLU involution measures to molecular subtype in adjacent normal tissues from breast cancers in Black women, a population with a higher incidence of aggressive TN breast cancer and the highest mortality rate due to breast cancer among all racial/ethnic groups in the USA. [1, 20]. As a similar trend of decreased TDLU involution in TN/CBP cancers as compared to luminal A cancers has been shown to exist across multiple racial/ethnic groups, it is possible that the biological mechanisms underlying these relationships may be similar across these groups.

TDLU involution has previously been associated with many breast cancer risk factors. Among healthy women, decreased TDLU involution has been observed for non-smokers compared to former and current smokers, women with family history of breast cancer, with younger ages at menarche, and with denser breasts [14, 21]. Among women diagnosed with BBD, decreased TDLU involution has been observed in those with lower BMI and younger age at first birth [15]. Older age and nulliparity have been associated with increased TDLU involution in studies of women with and without breast cancer [9, 14, 15].

Among the Black breast cancer patients in this study, we also observed relationships between risk factors and involution metrics in their adjacent normal breast tissues. Consistent with prior studies [14], we observed increased TDLU involution in older women, as expected, and nulliparous women. Previous studies in Black women and other populations evaluating heterogeneity in effects of risk factors on breast cancer subtypes have shown that parity may be protective against luminal A, while increasing risk for TN breast cancers [6, 22, 23]. Future research is needed to explore whether TDLU involution may play a role in mediating the effect of parity and age at last birth on breast cancer subtype.

Our study also revealed decreased TDLU involution in never smokers compared with former/current smokers in tissue from women with luminal A tumors, consistent with previous findings in healthy women [14]. Additionally, we found decreased TDLU involution in women with lower BMI, as observed in women diagnosed with BBD [15]. A recent pooled analysis investigating associations between breast cancer risk factors and molecular subtype found that increasing BMI was associated with increased risk of luminal A-like and luminal B-like tumors, but not TN tumors, in older women (≥ 55 years) [23]. In our study, decreased TDLU involution was seen in women with lower BMI in both luminal A and TN adjacent normal tissue after age adjustment. As smoking and higher BMI are both well-established risk factors for cancer, the relationship between these demographic factors and TDLU involution metrics may be more complex when the molecular subtype differences of the adjacent tumor tissue are taken into consideration.

We observed associations between measures of TDLU involution and clinical characteristics. Our results showed decreased TDLU involution in breast cancer cases with larger tumor size and lymph node invasion, two clinical characteristics associated with more aggressive breast cancer subtypes, including TN breast cancer [24]. Understanding this link between TDLU involution and clinical tumor characteristics is important as the intrinsic molecular subtypes are not only associated with distinct risk factor profiles, but also with breast cancer survival. Decreased TDLU involution related to these clinical features suggests that involution patterns may not only be determinants of breast cancer incidence, but could also be associated with clinical outcomes and survival. Although prior work in ER + breast cancer patients did not identify an association between pre-treatment TDLU involution and breast cancer-specific survival [25], additional work is warranted to elucidate the relationship between TDLU measures and survival across breast cancer molecular subtypes and racial and ethnic population groups.

Although obtaining quantitative TDLU measurements from H&E-stained tissue is often time-consuming, laborious, and subjective, currently limiting its clinical and prognostic utility, recent work has focused on automated approaches to obtain these measurements using digital pathology tools [26, 27]. These efforts not only speak to the importance of the relationships between TDLU involution, breast cancer risk factors and breast cancer risk, but have the potential to improve our ability to predict breast cancer risk objectively and on a larger scale.

Limitations of this study include the use of adjacent normal breast tissues from women diagnosed with breast cancer, preventing establishment of temporality of the observed associations. The relatively small sample size, especially for TN cancers, resulted in low power for some risk factor associations. Strengths of this study include the case-only study design for investigating etiologic heterogeneity between molecular breast cancer subtypes [28, 29], and the standardized characterization of quantitative TDLU metrics in Black women, an understudied population disproportionately affected by TN breast cancer.

## Conclusions

Overall, this study confirms and extends previous findings of an association of reduced TDLU involution with higher risk for TN compared with luminal A breast cancer to Black breast cancer patients. Associations between TDLU metrics and breast cancer risk factors suggest that TDLU involution may play an etiologic role in breast cancer carcinogenesis. More research is needed to understand how breast cancer risk factors, including environmental and social factors, influence TDLU involution and the mechanisms that mediate TDLU involution and risk of breast cancer subtypes. Further studies should also examine TDLU involution in normal and adjacent normal tissues from Black women and other racial and ethnic groups. Gaining a better understanding of the complex and heterogeneous etiology of breast cancer in Black women is paramount to addressing and reducing the health disparities that greatly affect this population.

## Supplementary Information

**Additional file 1: Supplemental Table 1.** Association between TDLU measures and breast cancer subtype among adjacent normal breast tissue slides^, adjusted for percent fat. **Supplemental Table 2.** Correlations between TDLU involution measures.

## Data Availability

The datasets used and/or analyzed during the current study are available from the corresponding author on reasonable request.

## Abbreviations

TDLU: Terminal duct lobular involution
TN: Triple-negative
BBD: Benign breast disease
CBP: Core basal phenotype
H&E: Hematoxylin and eosin
ER: Estrogen receptor
PR: Progesterone receptor
HER2: Human epidermal growth factor receptor 2
